# Real‐world experience of tyrosine kinase inhibitors in children, adolescents and adults with relapsed or refractory bone tumours: A Canadian Sarcoma Research and Clinical Collaboration (CanSaRCC) study

**DOI:** 10.1002/cam4.6515

**Published:** 2023-09-19

**Authors:** Hagit Peretz Soroka, Tushar Vora, Jonathan Noujaim, Nicolas Marcoux, Sarah Cohen‐Gogo, Thierry Alcindor, Caroline Holloway, Caroline Rodrigues, Hatim Karachiwala, Saima Alvi, Ursula Lee, Sylvia Cheng, Shantanu Banerji, Sapna Oberoi, Xiaolan Feng, Alannah Smrke, Christine Simmons, Albiruni Abdul Razak, Abha A. Gupta

**Affiliations:** ^1^ Division of Medical Oncology, Princess Margaret Cancer Centre University of Toronto Toronto Ontario Canada; ^2^ Division of Hematology/Oncology, Hospital for Sick Children University of Toronto Toronto Ontario Canada; ^3^ Division of Medical Oncology, Hôpital Maisonneuve Rosemont University of Montreal Montreal Quebec Canada; ^4^ Division of Hematology‐Oncology Centre Hospitalier Universitaire de Québec Quebec Canada; ^5^ Division of Medical Oncology McGill University Health Centre Montreal Quebec Canada; ^6^ Division of Radiation Oncology, BC Cancer University of British Columbia Vancouver British Columbia Canada; ^7^ Division of Medical Oncology, Cross Cancer Institute Alberta Health Services Edmonton Alberta Canada; ^8^ Division of Pediatric Hematology/Oncology Jim Pattison Children's Hospital Saskatoon Saskatoon Saskatchewan Canada; ^9^ Division of Medical Oncology, BC Cancer University of British Columbia Vancouver British Columbia Canada; ^10^ Division of Pediatric Hematology/Oncology/BMT B.C. Children's Hospital, BC Cancer Vancouver British Columbia Canada; ^11^ Department of Pediatric Hematology‐Oncology, CancerCare Manitoba Research Institute, Rady Faculty of Health Sciences University of Manitoba Winnipeg Manitoba Canada; ^12^ Division of Medical Oncology, Mount Sinai Hospital University of Toronto Toronto Ontario Canada

**Keywords:** cabozantanib, CanSaRCC, chondrosarcoma, Ewing sarcoma, osteosarcoma, regorafenib

## Abstract

**Objectives:**

We conducted a retrospective multi‐centre study to assess the real‐world outcome of regorafenib (REGO) and cabozantinib (CABO) in recurrent/refractory bone tumours (BTs) including osteosarcoma (OST), Ewing sarcoma (EWS) and chondrosarcoma (CS)/extra‐skeletal mesenchymal CS (ESMC).

**Methods:**

After regulatory approval, data from patients with recurrent BT (11 institutions) were extracted from CanSaRCC (Canadian Sarcoma Research and Clinical Collaboration) database. Patient characteristics, treatment and outcomes were collected. Progression‐free survival (PFS) and overall survival (OS) were estimated using the Kaplan–Meier method.

**Results:**

From July 2018 to May 2022, 66 patients received REGO or CABO; 39 OST, 18 EWS, 4 CS and 5 ESMC. Median age was 27.8 years (range 12–76); median starting dose was 60 mg for CABO (*n* = 37, range 40–60) and 120 mg for REGO (*n* = 29, range 40–160). Twenty‐eight (42.4%) patients required dose reduction: hand‐foot syndrome 7 (10.6%), nausea/vomiting 1 (1.5%), diarrhoea 1 (1.5%), 2 elevated LFTs (3%), elevated bilirubin 1 (1.5%) and mucositis 1 (1.5%). The median OS for patients with OST, EWS, CS and ESMC was 8.5 months (*n* = 39, 95% CI 7–13.1); 13.4 months (*n* = 18, 95% CI 3.4–27.2), 8.1 (*n* = 4, 95% CI 4.1–9.3) and 18.2 (*n* = 5, 95% CI (10.4–na), respectively. Median PFS for OST, EWS, CS and ECMS was 3.5 (*n* = 39, 95% CI 2.8–5), 3.9 (*n* = 18, 95% CI 2.1–5.9), 5.53 (*n* = 4. 95% CI 2.13–NA) and 11.4 (*n* = 5, 95% CI 1.83–14.7), respectively. Age, line of therapy, REGO versus CABO, or time from diagnosis to initiation of TKI were not associated with PFS on univariable analysis.

**Conclusion:**

Our real‐world data show that TKIs have meaningful activity in recurrent BT with acceptable toxicities when started at modified dosing. Inclusion of TKIs in earlier lines of treatment and/or maintenance therapy could be questions for future research.

## Abstract 2

Real‐world national CanSaRCC data support the use of regorafenib and cabozantinib in children and adults with relapsed bone sarcoma.

## 1 INTRODUCTION

Bone tumours account for less than 1% of diagnosed cancers every year. The most common primary bone tumours include osteosarcoma (OST), Ewing sarcoma (EWS) and chondrosarcoma (CS). The management of EWS and OS includes aggressive surgery and/or radiation in combination with chemotherapy. Surgery remains the only curative option for CS. Despite multimodality therapy, at least 30%–40% of patients will experience relapse of their disease. Patients with large or axial tumours and those with high burden of distant metastases at diagnosis have the highest risk of recurrence. There is a lack of effective treatment options for recurrent bone tumours and there remains an urgent unmet medical need.^1^, ^2^

Depending on location and timing of relapse, traditional approaches to second line therapy have been dominated by cytotoxic chemotherapies.^3^, ^4^ However, outcomes with cytotoxic chemotherapy remain poor and the recently published studies reporting the activity of tyrosine kinase inhibitors (TKIs) in patients with relapsed bone tumours were met with great hope.^5^, ^6^, ^7^, ^8^ Tyrosine kinases are key signalling proteins involved in cell growth and metastasis.^9^ Inhibition of these proteins have shown relevant implications regarding therapeutics in sarcoma.^10^

Cabozantinib (CABO) is a multi‐TKI targeting MET, VEGFR2, RET, KIT, FLT3, TIE2 and AXL, with important activity in several cancers such as renal cell carcinoma and prostate cancer.^11^, ^12^ In a phase II study involving children and adults with relapsed or advanced OST and EWS, partial responses were seen in 10 (26%; 95% CI 13–42) of 39 EWS patients and 5 (12%; 95% CI 4–26) of 42 OST patients^5^ at dose of 60 mg in adults and 40 mg/m^2^ in children, orally once daily in 28‐day cycles. Regorafenib (REGO) is another multi‐targeted TKI which was evaluated in a phase II study of 42 adults with relapsed OST. The initial dose of REGOwas 160 mg orally for Days 1–21 of a 28‐day cycle. Here, median progression‐free survival (PFS) was significantly improved among those who received REGO compared with placebo with a PFS of 3.6 months (95% CI, 2.0–7.6 months) versus 1.7 months (95% CI, 1.2–1.8 months), respectively (HR 0.42; 95% CI, 0.21–0.85; *p* = 0.017).^13^ Similar benefit compared to placebo was noted in a French study of 43 adults with OST.^8^ In the same study, a cohort of adults with CS also derived benefit from REGO with a PFS of 19.9 weeks compared with 8 weeks on placebo.^14^ Notably, both CABO and REGO offered patients an oral drug option with manageable toxicity and meaningful activity, addressing an area of significant clinical and patient need. Real‐world evidence for the use of these medications is not yet known.

Herein, we use a newly formed national database platform called CanSaRCC (Canadian Sarcoma Research and Clinical Collaboration) to report experience using CABO and REGO in children and adults with relapsed or refractory bone tumours to add real‐world evidence to these initial trials.

## 2 METHODS

After research ethics board approval at the following 11 institutions: The Hospital for Sick Children (Toronto), Princess Margaret Cancer Centre (Toronto), Mount Sinai Hospital (Toronto), BC Children's Hospital (Vancouver), BC Cancer (British Columbia), Hôpital Maisonneuve Rosemont (Montreal), Centre Hospitalier Universitaire de Québec (Québec City), the McGill University Health Centre (Montreal), Alberta Health Services (Edmonton), CancerCare Manitoba (Winnipeg), the Saskatchewan Health Authority (Saskatchewan), patient data were collated into the CanSaRCC database. Patients of all ages with documented relapsed or refractory OST, EWS or CS who received either CABO or REGO for at least 4 weeks were between July 2018 and May 2022 were included. All patients had measurable disease at the time of initiation of TKI therapy. Patients' demographics, disease and treatment details were then collected using a case report form and summarized using descriptive statistics. Treatment‐related toxicities were graded according to CTCAEv5. Response assessment was performed by local investigators. PFS and overall survival (OS) were estimated using the Kaplan–Meier method and compared using log‐rank test. PFS was calculated from the date of initiation of the TKI to the date of disease progression or death as determined by the local investigator. Disease progression or death from the disease was considered an event for PFS. OS was calculated from the date the patient started the TKI to the date of last follow‐up (including death). Clinical benefit rate (CBR) was defined as complete response (CR) plus partial response (PR) plus stable disease (SD) > 3 months. Univariable (UVA) analyses were conducted to examine the association of patient‐, disease‐ and treatment‐related variables with outcomes of PFS, OS and length of TKI therapy (TKI start to TKI end, if continuous then TKI start to last FU/death). We used student's *t*‐test/ANOVA and/or Wilcoxon rank/Kruskal–Wallis tests to compare means and medians between groups. Chi‐square test was used to compare categorical variables. For all analyses, *p* value <0.05 was considered statistically significant. Statistics were performed using SAS version 9.4.

## 3 RESULTS

### 3.1 Patient characteristics

Sixty‐six patients were included with a median age of 27.8 years (range 12–76) at the start of TKI, of whom, 20 (30%) were ≤ 18 years and 34 (51.5%) were male (see Table 1). The diagnoses were as follows: 39 (59.1%) OST, 18 (27.3%) EWS, 4 (6.1%) CS and 5 (7.6%) were extra‐skeletal mesenchymal CS (ESMC).

**TABLE 1:** Patient baseline characteristics.

		Regorafenib (*n* = 29)	Cabozantinib (*n* = 37)
Osteosarcoma (*n* = 39)	*n* Median age at TKI start (range)	25 25.1 (14.4–73.5)	14 21.5 (12.9–73)
	Age ≤ 18 years	10	4
	Male	9	9
	Previous lines of therapy, median (range)	1 (1–6)	2 (1–4)
	Location of primary Axial (*n*) Appendicular (*n*)	10 15	4 10
	Starting dose (mg)	120 (40–160)	50 (40–60)
Ewing sarcoma (*n* = 18)	*n* Median age at TKI start (range)	2 41.5 (20–63)	16 25 (12–61.8)
	Age ≤ 18 years	0	6
	Male	1	11
	Previous line of therapy, median (range)	2 (1–3)	2 (1–4)
	Location of primary Axial (*n*) Appendicular (*n*)	1 1	9 7
	Starting dose (mg)	120	60 (40–60)
Chondrosarcoma (*n* = 4)	*n* Median age at TKI start (range)	1 63.3	3 61.8 (58.6–76)
	Age (≤18)	0	0
	Male	0	2
	Previous lines of therapy, median (range)	2	1 (0–2)
	Location of primary Axial (*n*) Appendicular (*n*)	0 1	3 0
	Starting dose(mg)	120	40 (40 ‐ 60)
ECMS (*n* = 5)	*n* Median age at TKI start (range)	1 46.3	4 53.8 (23.9–69.4)
	Age (≤18)	0	0
	Male	0	2
	Previous lines of therapy, median (range)	2	2 (0–5)
	Location of primary Axial (*n*) Appendicular (*n*)	1 1	0 3
	Starting dose(mg)	120	40 (40–60)

**FIGURE 1:**
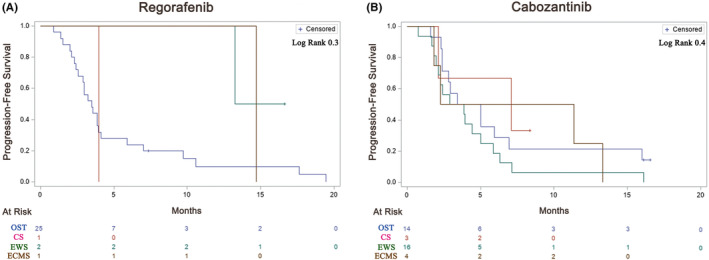
Progression‐free survival (PFS) of 66 patients with recurrent or refractory bone tumours treated either with regorafenib (A) or with cabozantinib (B). Osteosarcoma (Blue), chondrosarcoma (Red), Ewing's sarcoma (Green) and Extra‐skeletal mesenchymal CS (Brown).

The median OS for patients with OST, EWS, CS and ESMC were 8.5 months (*n* = 39, 95% CI 7–13.1), 13.4 months (*n* = 18, 95% CI 3.4–27.2), 8.1 months (*n* = 4, 95% CI 4.1–9.3) and 18.2 months (*n* = 5, 95% CI 10.4–na), respectively (Figure 1). The median PFS for patients with OST, EWS, CS and ESMC were 3.5 months (*n* = 39, 95% CI 2.8–5), 3.9 months (*n* = 18, 95% CI 2.1–5.9), 5.53 (*n* = 4. 95% CI 2.13–NA) and 11.4 (*n* = 5, 95% CI 1.83–14.7), respectively. (Figure 1).

### 3.2 Treatment, dose reductions and toxicity

#### 3.2.1 Cabozantinib

**FIGURE 2:**
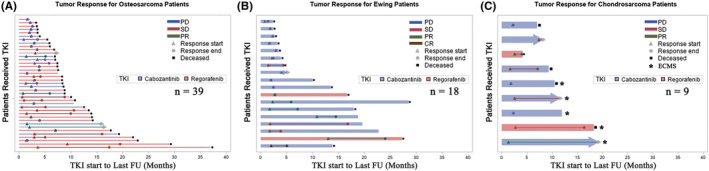
Swimmer plots and tumour response for patients treated either with regorafenib (pink bar) or with cabozantinib (light blue bar). (A) Osteosarcoma (*n* = 39), (B) Ewing's sarcoma (*n* = 18) and (C) chondrosarcoma (*n* = 9, ECMS—five out of nine). Bars ending with an arrow signify patients who are alive and continued response to TKI. A black square along the *y*‐axis signifies patients who are deceased. The *x*‐axis begins when the patient starts TKI and ends with patient's last follow‐up. Response start date, denoted by triangle, is the date of best response assessment. The response and date denoted by a circle is the date of treatment end. PD (progression disease); SD (stable disease); CR (complete response) can be identified within each bar and are denoted by blue, red, green and dark brown lines, respectively. For patients whose best response is PD, the response start and end date overlap.

**FIGURE 3:**
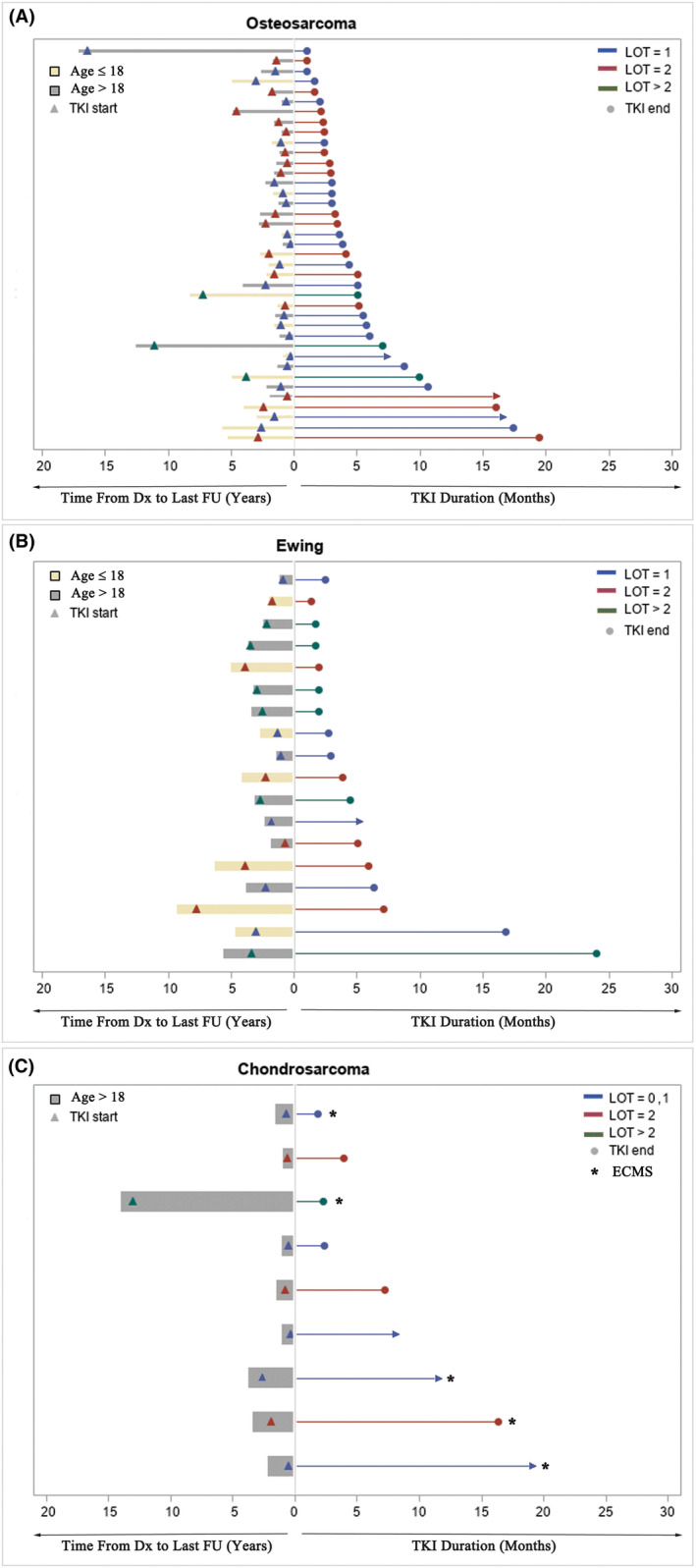
Swimmer plots of TKI duration, line of therapy (LOT) and tumour progression of children versus adults diagnosed with osteosarcoma (A), Ewing sarcoma(B), chondrosarcoma or ECMS (C). (A,B,C—right plot) ‐ The *x*‐axis begins when the patient starts TKI and ends on last day the patient received TKI (denoted by circle). LOT = 1, LOT = 2 and LOT >2 can be identified within each bar and are denoted by blue, red and green, respectively. (A,B,C—Left Plot) ‐ The *x*‐axis begins when the patient was diagnosed with bone tumour and ends on last follow‐up. Children (Age ≤ 18) and adults (Age ≥ 18) are denoted by yellow and grey lines, respectively. TKI start can be identified by triangle and denoted by blue (LOT = 1), red (LOT = 2) and green (LOT >2).

A total of 37 (56%) patients received CABO with a starting fixed dose of 40 mg (*n* = 17, 4 age ≤ 18 years) or 60 mg (*n* = 20, 6 age ≤ 18) once daily on a continuous basis. Dose reductions were required in 15 (40.5%) patients due to toxicity (Table 2). Dose reductions occurred in 7/17 (43.7%) for those who started at 40 mg and 8/20 (40%) for those who started at 60 mg. Four (10.8%) of these patients experienced Grade ≥3 toxicity with one having elevated LFTs, one having elevated bilirubin, one having hand‐foot syndrome and mucositis, and one having diarrhoea. Median duration of therapy for OST, EWS, CS and ECMS was 5 months (range 1.6–16.6), 3.4 (range 1.4–16.8), 7.23 (range 2.33–8.35) and 7 (1.84–19.32) months, respectively. Eleven patients (29.7%) received CABO for more than 6 months and six patients (16.2%) received CABO for more than 12 months (Figure 2). The age range of five of these six patients is 13–27 years, with one patient being 69 years old (Figure 3).

**TABLE 2:** Outcome of study population (*n* = 66).

		Regorafenib (*n* = 29)	Cabozantinib (*n* = 37)	*p*
Osteosarcoma *n* = 39	*n* Deceased (%)	25 23 (92)	14 11 (78.6)	ns
	Progression on TKI (%)	24 (96)	12 (85.7)	ns
	Best response			
	PD (%)	16 (64)	6 (42.9)	0.08
	Prolonged SD >3 months (%)	6 (24)	3 (21.4%)	
	PR (%)	1 (4)	4 (28.6)	
	CBR (%)	9 (36)	8 (57.1)	ns
	Dose reduction (%)	12 (48)	6 (42.8)	ns
	Median time from Diagnosis to TKI start (months) (range)	18 (3.3–197)	11.1 (4.6–86.3)	ns
	Median time from TKI start to best response (months) (range)	3 (0.9–9.3)	1.8 (0.6–3)	0.009
	Median length of therapy (months) (range)	3.6 (1–19.5)	5 (1.6–16.6)	ns
	Median time to progression months (range)	3.3 (0.9–19.4)	3.1 (1.6–16)	ns
Ewing sarcoma *n* = 18	*n* Deceased (%)	2 2 (100)	16 12 (75)	ns
	Progression on TKI (%)	1 (50)	16 (100)	0.004
	Best response			
	PD (%)	0	9 (56.2)	ns
	Prolonged SD >3 months (%)	0	2 (12.5)	
	PR (%)	2 (100)	3 (18.7)	
	CR (%)	0	1 (6.2)	
	CBR (%)	2 (100)	7 (43.7)	ns
	Dose reduction (%)	1 (50)	5 (31.3)	ns
	Median time from Dx to TKI start	27.7 (15.4–40)	28.6 (8–92.9)	ns
	Median time from TKI start to best response (months) (range)	7.9 (2.7–13)	2 (0.8–3.9)	0.08
	Median length of therapy (range)	13.4 (2.7–24)	3.4 (1.4–16.8)	ns
	Median time to progression (range)	13.24	3.4 (0.8–16)	ns
Chondrosarcoma *n* = 4	*n* Deceased (%)	1 1 (100%)	3 2 (66.7%)	na (not applicable sample size too small)
	Progression on TKI (%)	1 (100%)	2(66.7%)	na
	PD (%)	0	1 (33%)	na
	Prolonged SD >3 months (%)	0	1 (33%)	
	PR (%)	1 (100%)	0	
				na
	Dose reduction (%)	0	2 (66.7%)	na
	Median time from Dx to TKI start (range), mo	6.9	5.9 (4.4–8.9)	na
	Median time from TKI start to best response (months) (range)	2.56	2.14 (1.7–7.4)	na
	Median length of therapy	3.94	7.23 (2.3–8.35)	na
	Median time to progression (range)	4	4.6 (2.1–7.1)	
ECMS *n* = 5	*n* Deceased (%)	1 1 (100%)	4 1 (25%)	na
	Progression on TKI (%)	1 (100%)	4 (100%)	na
	PD (%)	0	2 (50%)	na
	Prolonged SD >3 months (%)	1 (100%)	1 (25%)	na
	PR (%)	0	1 (25%)	na
	Dose reduction (%)	0	2 (50%)	na
	Median time from Dx to TKI start (%)	22.5	20.2 (6.57–156.3)	na
	Median time from TKI start to best response (months) (range)	2.69	2.05 (1.3–2.6)	na
	Median length of therapy (range)	16.3	7 (1.8–19.3)	na
	Median time to progression (range)	14.7	6.8 (1.8–13.3)	na

Abbreviation: CBR, Clinical benefit rate—CR + PR + SD).

#### 3.2.2 Regorafenib

A total of 29 (44%) patients received REGO. Patients were treated with standard fixed dosing with a median starting dose of 120 mg (range 40–160 mg) administered for 21 days of a 28‐day cycle. For five patients with age ≤ 18, the starting dose was 40 mg to ensure tolerability and increased thereafter (Table 1). Dose reductions were required in 13 (44.8%) patients due to toxicity, of whom, 7 (24.1%) had Grade ≥3 (Table 2). Dose reductions occurred as follows: 1/5 (20%) at starting dose of 40 mg, 4/7 (57.1%) at 80 mg, 2/6 (33.3%) at 120 mg and 6/11 (54.5%) at 160 mg. Five patients had hand‐foot syndrome, one had nausea/vomiting and one presented with elevated bilirubin. The median duration of therapy for OST, EWS, CS and ECMS was 3.6 (range 1–19.5), 13.4 (range 2.7–24), 3.94 (*n* = 1) and 16.33 (*n* = 1) months, respectively. Eight patients received REGO for more than 6 months and four patients for longer than 12 months (Figure 2) including two patients aged 15 years, one aged 46 years and another aged 63 years.

Based on the data collected, no patients on CABO nor REGO discontinued treatment due to toxicity.

### 3.3 Outcome

Of 39 patients with OST, median PFS with CABO and REGO was 4.2 (*n* = 14, 95% CI 2.4–6.9) and 3.5 months (*n* = 25, 95% CI 2.6–4.1), respectively (Figure 1). CBR was 57.1% (*n* = 8/14, PR:4, SD: 4) for patients treated with CABO and 36% (*n* = 9/25, PR:1, SD: 8) with REGO. Objective responses (all PR) were observed in 4 (28.6%) to CABO and in 1 (PR) (4%) to REGO. The longest TKI treatment duration with CABO and REGO was 16.5 and 19.5 months, respectively. Nine patients (23%) had notable SD: *n* = 3, 6 months; *n* = 4, 6–12 months; *n* = 2, >12 months) with either REGO or CABO (Figures 2A and 3A).

Of 18 patients with EWS, median PFS with CABO and REGO was 3.4 months (*n* = 16, 95% CI 2–5) and 13.2 months (*n* = 2, 95% CI 13.2–n/a), respectively (Figure 1) although PFS for only 2 patients is uninterpretable. CBR was 43.8% (*n* = 7/16, CR: 1, PR:3, SD: 3) with CABO and 100% (*n* = 2/2, all PR) with REGO. Objective responses were seen in 4 patients (CR: 1, PR: 3) (25%) with CABO and in two patients (PR only) (100%) with REGO. The longest duration of therapy with CABO and REGO was 16.8 and 24 months, respectively. One patient had notable SD lasting >12 months) (Figures 2B and 3B).

Of five patients with ECMS, the median PFS with CABO and REGO are 6.81 (*n* = 4, 95% CI 1.83–13.3) and 14.7 (*n* = 1), respectively. Of four patients with CS, the median PFS with CABO and REGO is 7.09 (*n* = 3, 95% CI 2.13–na) and 3.97 (*n* = 1), respectively (Figure 1). Responses in CS were as follows: PR (*n* = 1, REGO), prolonged SD (*n* = 1, CABO). Responses in ECMS were as follows: PR (*n* = 1, CABO), prolonged SD (*n* = 1, CABO; *n* = 1, REGO). The longest duration of therapy with CABO and REGO was 19.3 and 16.5 months, respectively (both ECMS). Three patients had notable SD: *n* = 1, <6 months; *n* = 1, 6–12 months; *n* = 1, >12 months) (Figures 2C and 3C) (all ECMS). Univariable analysis failed to show any factors that were associated with response including age, line of therapy, sex, location of primary, tumour type, REGO versus CABO, or time from initial diagnosis to TKI start.

There were two exceptional responders in our cohort. Case 1 is that of a 27‐year‐old female with OST, who suffered a relapse 12 years following her initial diagnosis (at which time she was treated with MAP chemotherapy) with an intracardiac mass causing severe right ventricular outflow tract obstruction and bilateral lung metastases. After urgent cardiac debulking, she received 5 cycles of ifosfamide/etoposide with good response. She was started on REGO due to persistent small lung lesions (largest 1.6 × 1.1 cm, 1.0 × 0.9 cm) and remains on drug without progression for 16.1 months at the data cut off for study. The second case is that of an 18‐year‐old male with OST who developed bilateral small volume pulmonary metastases (largest 1.1 × 0.6 cm) less than 2 years from primary diagnosis, who started CABO at first relapse and remains on drug without progression for 16.6 months.

## 4 DISCUSSION

Using the recently established infrastructure of a national sarcoma database, herein we present meaningful real‐world activity data of REGO and CABO in children, adolescents and adults with relapsed or refractory bone tumours. Oral TKIs offer patients a viable alternative to cytotoxic chemotherapy offering reprieve from alopecia, nausea and cytopenia, and in select cases, is associated with long term disease stability. Patients either started TKI at doses lower than previously published or required dose reductions highlighting the lack of clarity around the ideal therapeutic window of TKI in bone tumours.

The outcome for patients with advanced bone tumours has remained stagnant over the last 40 years,^15^ and although the essential components of induction therapy including definitive local control and some amount of cytotoxic chemotherapy remain, dose intensification or addition of other chemotherapy agents have failed to offer solutions.^16^ Considering prolonged disease stability observed in some patients, especially for those in whom TKI was started early, offering TKI in first relapse may be an important strategy to consider moving forward. Due to low power, we were unable to prove statistical significance that line of therapy impacted outcome. The PFS seen in our cohort is comparable to those described in the seminal studies of REGO and CABO,^5^, ^8^, ^13^, ^14^ although our series excluded patients who were on drug for less than 4 weeks and events were not centrally reviewed. CBR is an important outcome to patients reflecting the ability to stay on life prolonging treatment. Thus, TKI for maintenance treatment to delay subsequent progression may be important to consider in bone tumours, although this was not done in our current series. Previously, maintenance therapy with an mTOR inhibitor, ridaforolimus in adults with soft tissue or bone sarcoma was explored, although with minimal clinical benefit.^17^ In comparison, maintenance chemotherapy does in fact improve survival in soft tissue rhabdomyosarcoma.^18^ Further studies are needed to precisely identify the role of maintenance TKI therapy in bone tumours and we look forward to the results of an ongoing trial evaluating CABO maintenance therapy in high‐risk sarcoma (clinicaltrials.gov identifier NCT05135975

This study included patients of all ages, and we observed variability in dosing practices across ages and institutions. Many paediatric patients were treated with flat dosing instead of dosing per m^2^. Moreover, the starting dose of CABO was only 40 mg in half of the adult patients (13/30) and median dose of REGO was 120 mg for all patients. In four paediatric patients, REGO was started at 40 mg and dose slowly increased to tolerance. We suggest exploring a ‘go slow’ approach to maximize tolerance to drug, a strategy similar to that implemented in other cancers.^19^

CS is a rare form of bone cancer that is mainly treated with surgery and is generally unresponsive to traditional chemotherapy options.^20^ Targeted inhibition of the receptor pathway has been suggested to delay tumour growth supporting the rational to study TKIs in this sarcoma subset.^21^ In the REGOBONE trial, patients with CS responded to REGO even after having received chemotherapy for relapsed disease. In our series, five of nine patients with CS had ESMC (four confirmed HEY1‐NCOA2 gene fusion) who experienced prolonged disease control with TKI, similar to prior reports.^22^ Interestingly, the main downstream targets of HEY1‐NCOA2 include PDGFRA, PDGFRB and BCL2 perhaps contributing to the sensitivity to TKI.^23^

Other TKIs have been examined in recurrent/refractory bone tumours including sorafenib in OST (PFS 4 (95% CI 2–5)),^24^ and sorafenib plus everolimus (significant toxicity).^25^, ^26^ In the current study, we did not evaluate the outcome of patients who may have received sorafenib for OST.

This study was conducted using a national database (Canadian Sarcoma Research and Clinical Collaboration) that permitted the capture of data for patients with sarcoma diagnosed across a wide geographical area and facilitated meaningful analysis on a rare subset of patients. This infrastructure that includes pre‐defined data elements, quality check and longitudinal follow‐up offers many opportunities to collect real‐world data to help supplement clinical trials. This is the first study conducted through the CanSaRCC national collaboration of 11 sites across Canada permitting the unique opportunity to increase the sample size of patients with rare bone sarcomas receiving TKIs as a therapeutic intervention. In this regard, institutions were permitted to offer imaging evaluations at their own discretion and provided their own assessment of response and toxicity to drug. Therefore, our description of PFS is made within these limitations. We also did not collect details on disease burden prior to starting therapy and thus could not comment on whether TKI slowed the pace of disease progression. Furthermore, despite this national effort, the rarity of relapsed and refractory bone tumours precluded our ability to identify any patient factors that may predict response to TKI. Other limitations to this study include: (1) lack of information on the timing of when dose reductions occurred (first course or later), (2) no information on need for additional medications to treat side effects of TKIs and (3) the absence of information on whether the use of the TKI contributed to the ability to perform local control. Despite these limitations, further exploration of tumour biomarkers predicting response to anti‐angiogenic therapy in bone tumours is of utmost interest for future projects.

In summary, we demonstrate meaningful real‐world outcomes using TKI in children, adolescents and adults with relapsed/refractory bone tumours. Future trials should consider including TKIs as maintenance treatment. More national and international joint collaborations through sarcoma‐oriented databases such as CanSaRCC will help to share precise data and facilitate interdisciplinary clinical and translational research in sarcoma.

## AUTHOR CONTRIBUTIONS

**Hagit Peretz‐Soroka:** Conceptualization (equal); data curation (lead); formal analysis (lead); methodology (lead); project administration (lead); resources (lead); validation (lead); writing – review and editing (equal). **Tushar Vora:** Data curation (equal). **Jonathan Noujaim:** Conceptualization (equal); data curation (equal); writing – review and editing (equal). **Nicolas Marcoux:** Data curation (equal); writing – review and editing (equal). **Sarah Cohen‐Gogo:** Data curation (equal); writing – review and editing (equal). **Thierry Alcindor:** Data curation (equal); writing – review and editing (equal). **Caroline Holloway:** Data curation (equal); writing – review and editing (equal). **Caroline Rodrigues:** Data curation (equal); formal analysis (equal); validation (equal); writing – review and editing (equal). **Hatim Karachiwala:** Data curation (equal); writing – review and editing (equal). **Saima Alvi:** Data curation (equal); writing – review and editing (equal). **Ursula Lee:** Data curation (equal); writing – review and editing (equal). **Sylvia Cheng:** Data curation (equal); writing – review and editing (equal). **Shantanu Banerji:** Data curation (equal); writing – review and editing (equal). **Sapna Oberoi:** Data curation (equal); writing – review and editing (equal). **Xiaolan Feng:** Data curation (equal); writing – review and editing (equal). **Alannah Smrke:** Data curation (equal); writing – review and editing (equal). **Christine Simmons:** Data curation (equal); writing – review and editing (equal). **Albiruni R.A. Razak:** Data curation (equal); methodology (equal); writing – review and editing (equal). **Abha Gupta:** Conceptualization (lead); formal analysis (equal); methodology (lead); project administration (equal); resources (lead); supervision (lead); writing – original draft (lead); writing – review and editing (lead).

## FUNDING INFORMATION

This work was supported by CanSaRCC.

## CONFLICT OF INTEREST STATEMENT

None of the authors have any conflicts of interest to disclose.

## ETHICS STATEMENT

Ethics approval was obtained through University Health Network which supports the consortium agreement with CanSaRCC permitting multi‐institutional data and the lead site of this project.

## Data Availability

Data sharing is not applicable to this article as no new data were created or analyzed in this study.
